# When Appearances Deceive: A Case Report of Neurocysticercosis Masquerading As Tuberculoma

**DOI:** 10.7759/cureus.103762

**Published:** 2026-02-17

**Authors:** Hrithik Dakssesh Putta Nagarajan, Keerthivasan Selvanathan, Tejashvi Rameshkumar, Nitish Thirugnanasambandam, Md Ramij Biswas

**Affiliations:** 1 Department of Internal Medicine, Madurai Medical College, Madurai, IND; 2 Department of Internal Medicine, Krishnaswamy Ayyar Paramasivam (K.A.P. Viswanatham Government Medical College, Tiruchirappalli, IND; 3 Department of Internal Medicine, Dr RK Diabetic Foot and Podiatry Institute, Chennai, IND; 4 Department of Internal Medicine, Rajshree Medical Research Institute, Bareilly, IND

**Keywords:** case report, epilepsy, neurocysticercosis, tapeworm, tuberculoma

## Abstract

Neurocysticercosis (NCC) is a predominant cause of acquired epilepsy globally and often resembles cerebral tuberculoma in neuroimaging, particularly in regions where tuberculosis is prevalent. Diagnostic challenges are exacerbated in resource-limited settings due to financial limitations, restricted access to advanced diagnostic tools, and insufficient longitudinal medical records. We report a case of a 17-year-old immunocompetent female with recurrent generalized seizures who was repeatedly misdiagnosed with cerebral tuberculoma over an eight-year period and treated with multiple courses of anti-tubercular therapy based solely on imaging findings. During the current evaluation, magnetic resonance imaging identified a solitary, small, non-enhancing T2 hypointense lesion with surrounding edema in the right frontal lobe, prompting a differential diagnosis of NCC versus tuberculoma. Negative QuantiFERON-TB Gold (QFT-G, Cellestis Limited, Carnegie, Victoria, Australia) testing, normal chest radiography, absence of systemic tuberculosis, and prior extensive exposure to anti-tubercular therapy favored NCC. Treatment with albendazole and corticosteroids resulted in complete seizure control without recurrence. This case underscores the necessity for meticulous clinic-radiological correlation and increased diagnostic vigilance to prevent misdiagnosis and unnecessary treatment, particularly in resource-constrained environments.

## Introduction

Neurocysticercosis (NCC), a helminthic infection of the central nervous system, is a significant etiological factor for acquired epilepsy worldwide. This condition arises from infection with the tapeworm, *Taenia solium*, and manifests with a range of neurological symptoms contingent upon the number and location of the lesions and the intensity of the host’s immune response against the parasites. Neuroimaging is pivotal in the diagnostic process; however, radiological findings can be difficult to differentiate from other space-occupying intracranial lesions [1]. NCC accounts for up to 30% of epilepsy cases in endemic regions, underscoring its significant public health burden [2].

Tuberculomas are the commonest form of intracranial parenchymal tuberculosis (TB) [3]. NCC and tuberculoma exhibit considerable overlap in clinical, imaging and epidemiologic characteristics [4]. This diagnostic ambiguity may lead to misclassification and inappropriate initiation of therapy, resulting in unnecessary treatment exposure and delayed clinical improvement.

In this case report, we describe a case of NCC in a 17-year-old immunocompetent female who was repeatedly misdiagnosed as having cerebral tuberculoma based on neuroimaging findings alone. This case highlights the critical importance of comprehensive clinical evaluation, meticulous radiological analysis, and the inclusion of parasitic infections in the differential diagnosis of intracranial lesions, especially in resource-limited settings.

## Case presentation

A 17-year-old female was brought into the emergency room with complaints of seizure-like activity involving all four limbs, followed by postictal confusion. She regained consciousness shortly after admission. The patient reported a history of recurrent seizures and a prior diagnosis of extrapulmonary TB involving the brain, for which she had completed two courses of standardized anti-tubercular therapy (ATT) according to national guidelines in India.

A review of available medical records revealed a magnetic resonance imaging (MRI) scan of the brain performed one year earlier that reportedly showed a 5-mm non-enhancing T2 hypointense nodule with mild perilesional edema in the right frontal region; however, the original images were unavailable for review. General physical and systemic examinations, as well as vital parameters, were within normal limits. There were no focal neurologic deficits.

**Figure 1:**
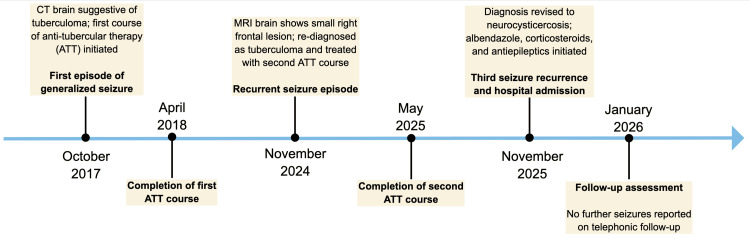
Clinical Timeline of Recurrent Seizures and Diagnostic Reassessment Chronological summary of seizure episodes, neuroimaging findings, treatment with anti-tubercular therapy, diagnostic reassessment in November 2025, and clinical outcome on follow-up.

The first seizure episode had occurred eight years earlier, at which time non-contrast computed tomography (CT) of the brain reportedly demonstrated a small right frontal nodular lesion, interpreted as suggestive of cerebral tuberculoma; however, the original images could not be retrieved for review, and the patient subsequently completed a six-month course of ATT. She was started on carbamazepine, which was discontinued two years later following seizure remission. A second seizure episode occurred one year prior to the current presentation, prompting a repeat MRI and a renewed diagnosis of tuberculoma, for which she again received six months of ATT. Carbamazepine was restarted and subsequently discontinued eight months later due to the absence of further seizures. Despite repeated ATT, the recurrence of seizures raised concerns regarding the accuracy of the initial diagnosis. A timeline of clinical events is summarized in Figure 1.

The patient was loaded with Injection fosphenytoin 800 mg, followed by Injection phenytoin 100 mg intravenously three times daily, and Injection levetiracetam 500 mg intravenously twice daily. Injection lorazepam 3 mg intravenously was prescribed as needed for breakthrough seizures, in accordance with institutional protocol.

**Figure 2:**
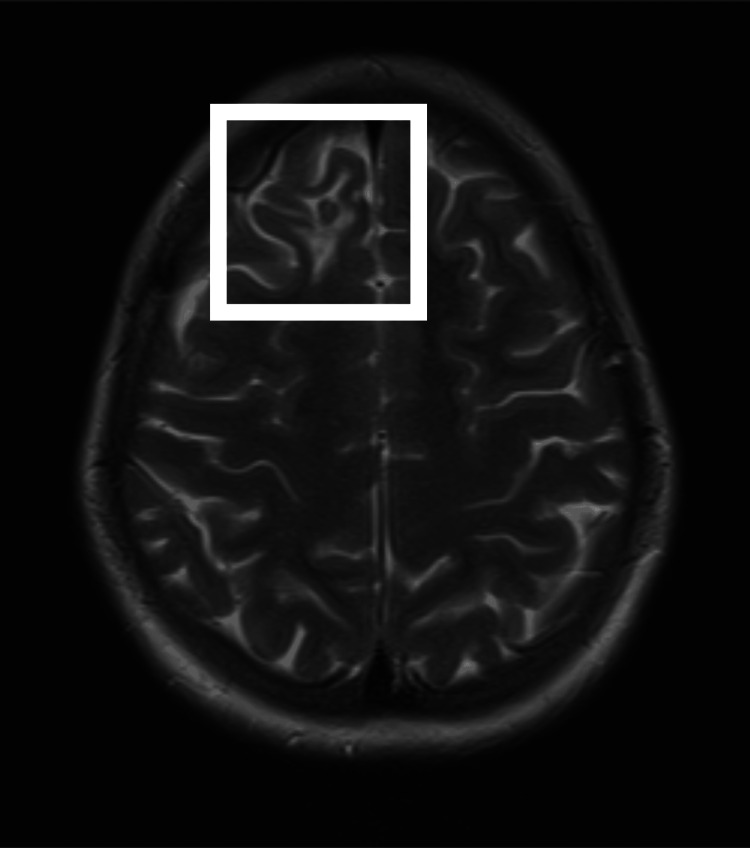
MRI of the Brain Showing Solitary Parenchymal Lesion Axial T2-weighted magnetic resonance imaging of the brain demonstrating a small, non-enhancing T2 hypointense lesion in the right parasagittal anterior frontal lobe, with surrounding hyperintense perilesional edema, suggestive of a solitary parenchymal neurocysticercosis lesion. The lesion is highlighted within the white box.

**Figure 3:**
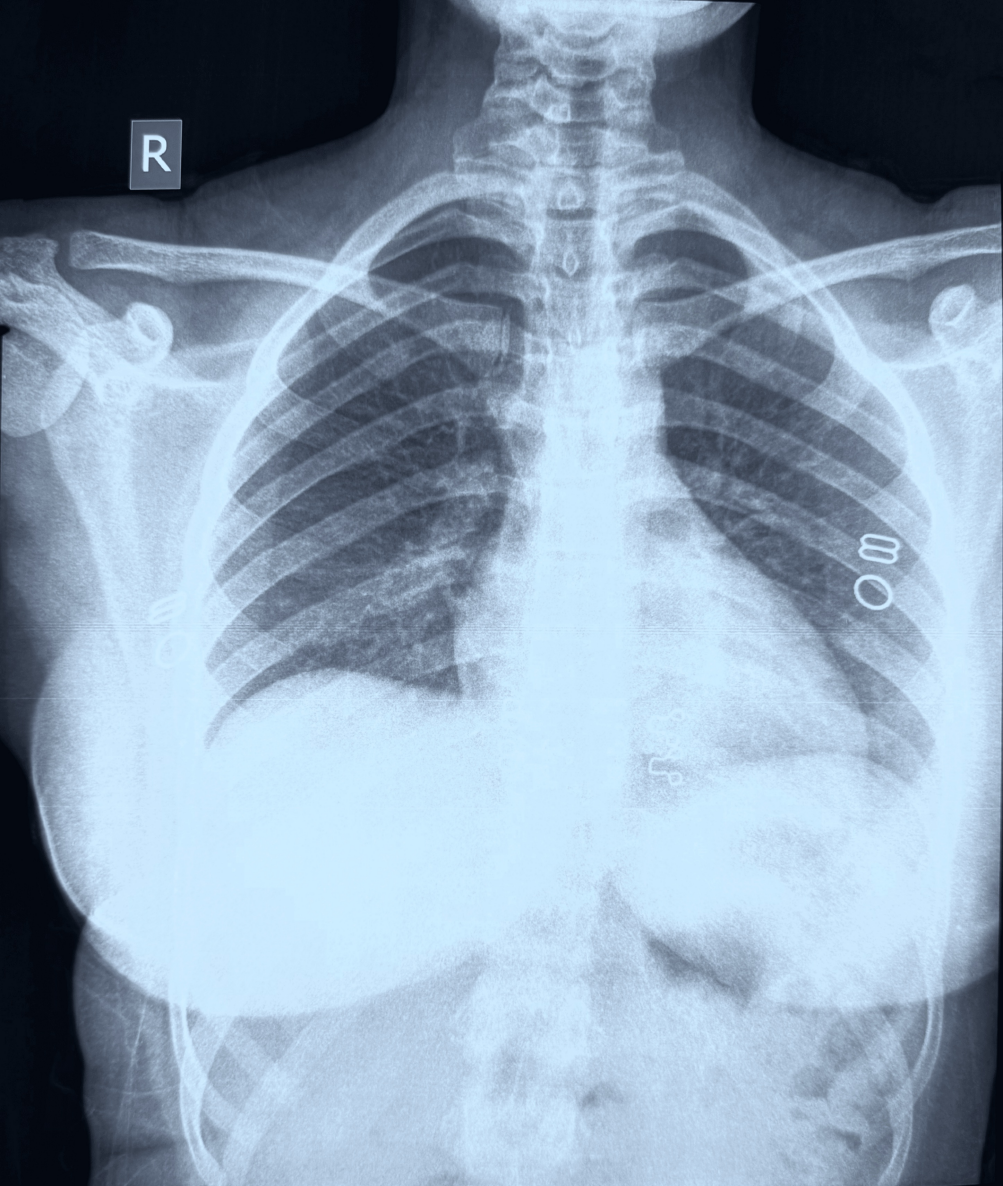
Chest X-ray Showing No Evidence of Pulmonary Tuberculosis Posteroanterior chest radiograph demonstrating normal lung fields with no focal consolidations, cavitary lesions, nodules, or lymphadenopathy, and no radiological evidence of active or healed pulmonary tuberculosis.

Neurology and neurosurgery consultations were obtained, and an MRI of the brain with contrast was performed. Axial T2-weighted MRI revealed a 6 × 5 mm non-enhancing hypointense lesion with surrounding edema in the right parasagittal anterior frontal lobe (Figure 2). The differential diagnosis included NCC and tuberculoma. Chest radiography was normal (Figure 3), and QuantiFERON-TB Gold (QFT-G, Cellestis Limited, Carnegie, Victoria, Australia) testing was negative. Given the absence of systemic TB, negative TB testing, and multiple prior courses of ATT, NCC was considered more likely than tuberculoma.

Serological testing for NCC could not be performed due to limited availability and financial constraints, representing a limitation of this report. The patient was treated with Injection dexamethasone 8 mg intravenously twice daily for five days to address perilesional edema, along with Tablet albendazole 400 mg orally twice daily for 10 days [5]. She was discharged on Tablet levetiracetam 500 mg orally three times daily.

On telephonic follow-up, the patient reported no further seizure episodes. A long-term plan for gradual tapering and discontinuation of antiepileptic therapy was discussed. Follow-up neuroimaging could not be performed due to logistical and financial limitations.

## Discussion

**Figure 4:**
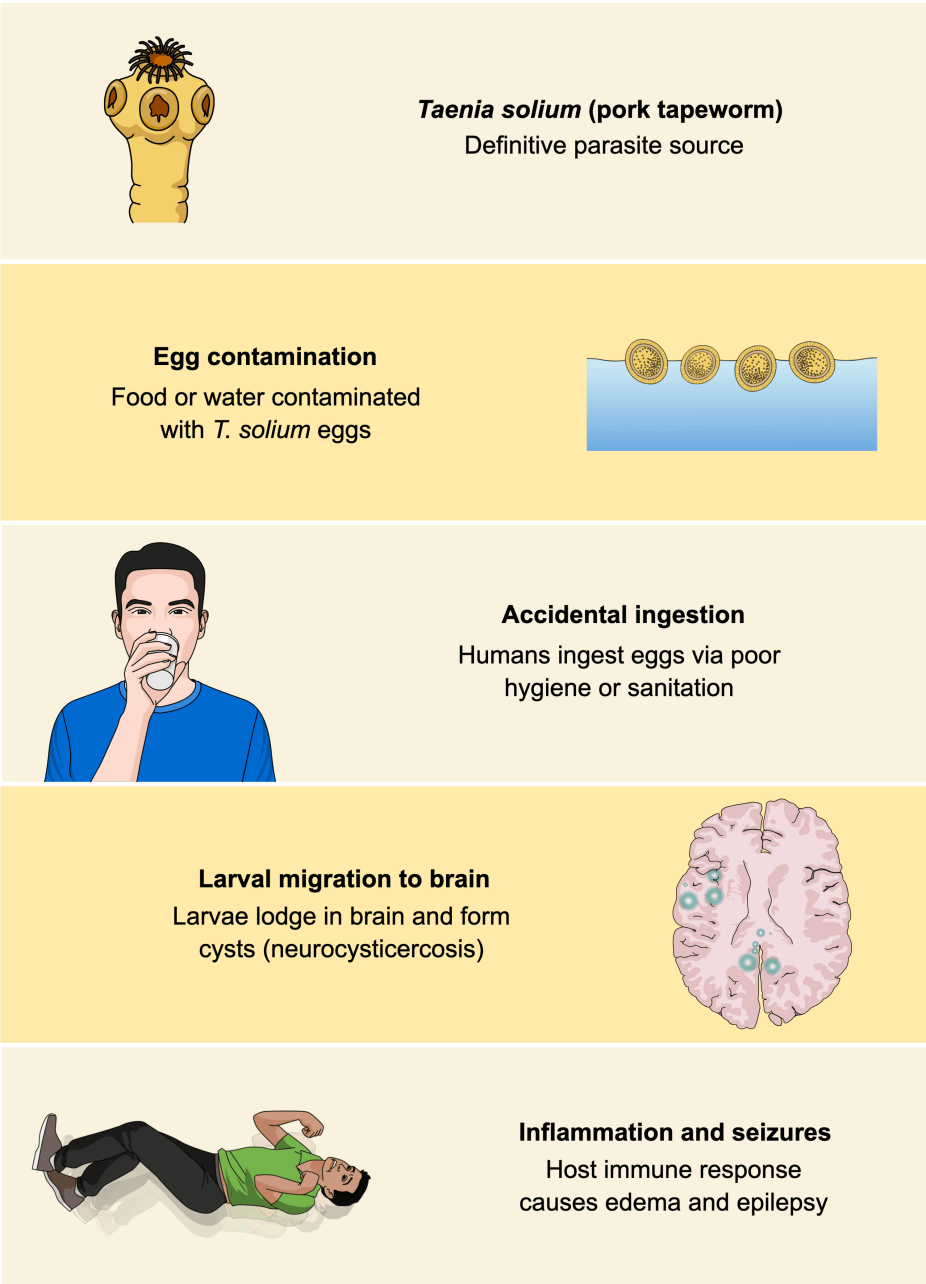
Graphical Representation of the Pathogenesis of Neurocysticercosis Leading to Seizures Schematic representation of the life cycle of *Taenia solium* and the development of neurocysticercosis, highlighting egg ingestion, cerebral cyst formation, and inflammation-mediated seizures. Image created by the authors based on *Del Brutto OH* [1].

NCC is primarily found in countries, like India, with poor sanitary infrastructure and improper slaughterhouse services. Our patient presented with a seizure, which is the most common presentation of NCC, occurring in about 80% of symptomatic patients. Other presentations include severe headache (38%), focal deficits (16%), and signs of increased intracranial pressure (12%) [6,7]. Figure 4 is included to provide contextual understanding of the pathophysiology of NCC for readers unfamiliar with the disease process.

Cerebral tuberculoma and NCC frequently resemble neoplastic lesions on neuroimaging. NCC and tuberculoma exhibit numerous similarities in both clinical presentation and neuroimaging findings, complicating their differentiation, which is nonetheless essential. Additionally, NCC and tuberculoma share epidemiological characteristics, further complicating the diagnostic process. Classically, cerebral tuberculoma presents as a solitary lesion, whereas NCC more commonly manifests with multiple lesions; however, solitary NCC lesions can significantly contribute to diagnostic confusion, as demonstrated in the present case [8].

In this patient, a neoplastic etiology was deemed less probable due to the lesion’s small size, lack of contrast enhancement, absence of interval growth over several years, disproportionate perilesional edema, and isolated seizure presentation without progressive neurological deficits. Furthermore, a favorable clinical response to antiparasitic therapy further reinforced the exclusion of a neoplastic etiology.

While several reports describe cerebral tuberculoma being misdiagnosed as NCC, reports of NCC being repeatedly misdiagnosed as tuberculoma are exceedingly rare [4,8]. In one such report, NCC was misdiagnosed as tuberculoma in a patient with multiple intracranial lesions, and ATT was not initiated [9]. In contrast, our patient had a solitary lesion and was repeatedly treated with ATT, further distinguishing our case.

The lack of seizure resolution following the initial six-month regimen of ATT should have prompted an early diagnostic reassessment, including the exclusion of TB and consideration of parasitic causes. Continued reliance solely on imaging, without sufficient clinico-radiological correlation, led to prolonged exposure to ATT and delayed definitive treatment. This issue was exacerbated by limited access to previous imaging and fragmented longitudinal clinical documentation, which restricted meaningful temporal comparisons. This case underscores how anchoring bias in TB-endemic regions, combined with incomplete medical records in resource-limited settings, can contribute to delayed diagnostic reassessment, highlighting the importance of systematic re-evaluation when clinical response is inadequate.

Magnetic resonance spectroscopy (MRS) is yet another important test that can aid in differentiating between NCC and tuberculoma. MRS is a technique that can measure the chemical information from a selected region within the tissue of interest [10]. The MRS study of NCC shows a combination of elevated lactate, alanine, succinate, and choline levels and reduced levels of N-acetyl aspartate and creatine in the lesion [11]. On the other hand, an MRS study of tuberculoma shows the presence of a dominant lipid peak and near absence of other metabolites because a major part of *Mycobacterium tuberculosis*, particularly its wall, is known to contain lipids [12]. However, MRS was not performed on this patient due to limited availability at the treating center.

Serological tests such as enzyme-linked immunosorbent assay (ELISA) and electro-immunotransfer blot (EITB) assay may assist in diagnosing NCC but have reduced sensitivity in cases with solitary lesions. The EITB assay, while highly specific, yields false-negative results in more than half of patients with single intracranial lesions, further limiting its utility in cases such as ours [13]. Key comparative aspects of NCC and cerebral tuberculoma are summarized in Table 1.

**Table 1:** Comparative overview of neurocysticercosis and cerebral tuberculoma. Data summarized from previously published literature [1,6–8,11–13]. EITB: Electro-immunotransfer blot; ELISA: Enzyme-linked immunosorbent assay; MRI: Magnetic resonance imaging

Feature	Neurocysticercosis (NCC)	Cerebral Tuberculoma
Etiology (causative organism)	Parasitic infection caused by *Taenia solium* (larval cysts)	Mycobacterial granulomatous infection caused by *Mycobacterium tuberculosis*
Typical lesion number	Usually multiple	Usually solitary
MRI spectroscopy	Elevated lactate, alanine, succinate, choline; reduced N-acetyl aspartate and creatine	Dominant lipid peak with relative absence of other metabolites
Serology/ancillary tests	EITB/ELISA may assist in diagnosis	QuantiFERON-TB Gold (QFT) may assist in diagnosis
Systemic features	Usually absent	May be associated with systemic tuberculosis
Chest imaging	Typically normal	May show pulmonary or lymph node involvement
Treatment	Improves with antiparasitic therapy ± corticosteroids	Responds to anti-tubercular therapy
Seizure presentation	Common initial manifestation	Less common as isolated presentation

This case emphasizes the risk of unnecessary prolonged ATT exposure, potential drug-related toxicity, and delayed seizure control, particularly in resource-constrained settings. NCC represents a significant public health concern that necessitates serious disease control efforts, with the ultimate objective of eradication. NCC could potentially be eradicated through the implementation of several strategies. These include improvement in sanitation, better pig-rearing practices, mass drug treatment of pigs, and targeted or mass treatment of humans [14]. Additionally, a porcine vaccine targeting tapeworm antigens has been developed and is proven to be effective [15].

## Conclusions

NCC and cerebral tuberculoma present a considerable diagnostic challenge due to their overlapping clinical, radiological, and epidemiological features, particularly in regions where TB is endemic. This challenge is magnified in resource-limited settings, where financial constraints, restricted access to advanced diagnostics, and inadequate longitudinal medical documentation hinder accurate diagnosis. This case illustrates how NCC can be repeatedly misdiagnosed as tuberculoma, resulting in unnecessary exposure to prolonged ATT and delayed definitive treatment. Maintaining a high index of suspicion for NCC, even in patients with solitary intracranial lesions, is essential to reduce diagnostic delays, minimize treatment-related morbidity, and improve patient outcomes.
